# Perceived Barriers and Facilitators to Physical Activity Engagement Among Cancer Survivors: A Qualitative Study

**DOI:** 10.3390/cancers18050817

**Published:** 2026-03-03

**Authors:** Gaurav Kumar, Priyanka Chaudhary, Apar Kishor Ganti, Jungyoon Kim, Lynette M. Smith, Dejun Su

**Affiliations:** 1 TSET Health Promotion Research Center, Stephenson Cancer Center, The University of Oklahoma Health Campus, Oklahoma City, OK 73104, USA; 2 Pennington Biomedical Research Center, Louisiana State University, Baton Rouge, LA 70808, USA; priyanka.chaudhary@pbrc.edu; 3 Internal Medicine Division of Oncology & Hematology, Fred and Pamela Buffett Cancer Center, University of Nebraska Medical Center, Omaha, NE 68198, USA; aganti@unmc.edu; 4 VA Nebraska Western Iowa Health Care System, 4101 Woolworth Avenue, Omaha, NE 68105, USA; 5 Department of Health Services Research & Administration, College of Public Health, University of Nebraska Medical Center, Omaha, NE 68198, USA; jungyoon.kim@unmc.edu; 6 Department of Biostatistics, College of Public Health, University of Nebraska Medical Center, Omaha, NE 68198, USA; lmsmith@unmc.edu; 7 Department of Family Medicine and Community Health, School of Medicine and Public Health, University of Wisconsin-Madison, Madison, WI 53705, USA; dejun.su@fammed.wisc.edu

**Keywords:** cancer survivorship, barriers, facilitators, recommendation, physical activity, qualitative research

## Abstract

**Background:** Although physical activity (PA) offers substantial physical and psychosocial benefits, engagement remains suboptimal among cancer survivors. A theory-informed understanding of survivors’ perceived barriers, facilitators, and recommendations is needed to inform patient-centered PA about survivorship interventions. **Objective:** This study aimed to explore perceived barriers, facilitators, and recommendations for PA engagement among adult cancer survivors using the Theoretical Domains Framework (TDF). **Methods:** A phenomenological qualitative design was used. Eighteen cancer survivors from Nebraska participated in semi-structured interviews via Zoom or telephone. Semi-structured interviews (guided by open-ended questions with flexibility for probing) were transcribed verbatim, imported into MAXQDA 2024, and analyzed using TDF to identify themes and subthemes. **Results:** Three overarching themes emerged: barriers, facilitators, and recommendations related to PA engagement. Barriers included individual factors (low motivation and self-efficacy, limited awareness of PA guidelines, time constraints, and physical limitations due to treatment and comorbidities), social factors (limited support from family, friends), clinical factors (limited PA guidance from healthcare providers), and environmental factors (restricted access to resources and unfavorable weather). Facilitators included individual factors (PA knowledge, motivation, goals, and health benefits), social factors (support from family, friends), and clinical factors (encouragement from healthcare providers), and environmental factors (favorable weather and available community PA resources). Recommendations emphasized the need for tailored education, supportive counseling, and structured PA programs within survivorship care. **Conclusions:** Cancer survivors described multilevel determinants of PA engagement across individual, social, and environmental contexts. Findings highlight the importance of theory-informed, patient-centered strategies that enhance PA guideline awareness, strengthen social and clinical support, and improve access to community resources to promote sustained PA during cancer survivorship.

## Simple Summary

Engaging in physical activity can promote better health, emotional well-being, and quality of life among cancer survivors, yet many survivors struggle to stay active during and after treatment. In this qualitative study, semi-structured interviews were conducted with 18 adult cancer survivors from Nebraska to explore experiences with physical activity, including what makes it difficult, what helps, and what additional support they need. Survivors described barriers such as treatment-related symptoms, low motivation, limited awareness of physical activity guidelines, lack of support from healthcare providers, and challenges related to weather and access to exercise resources. Facilitators included personal motivation, perceived health benefits, encouragement from family and friends, and access to community-based physical activity programs. Survivors emphasized the need for tailored education, supportive counseling from healthcare providers, and structured physical activity programs integrated into survivorship care. These findings highlight opportunities to develop patient-centered strategies to better support physical activity among cancer survivors.

## 1. Introduction

Cancer is a significant public health concern in the United States, ranking as the second leading cause of mortality and presenting substantial challenges to modern medicine [1,2]. In 2025, an estimated 2,041,910 new cancer cases and 618,120 cancer deaths are projected in the United States [1]. Due to advances in screening and treatment, survivorship has increased substantially, with approximately 18.6 million individuals currently living with a history of cancer, a number projected to exceed 22 million by 2035 [1]. As survivorship continues to rise, there is increasing recognition of the need to address long-term and supportive care needs across the survivorship continuum [3,4,5,6].

Despite advancements in cancer prognosis, a substantial proportion of cancer survivors continue to experience persistent, or late-onset effects related to cancer and its treatment. These include fatigue, anxiety, depression, peripheral neuropathy, and declines in strength and cardiorespiratory fitness, all of which can negatively impact daily functioning and the overall quality of life [7,8,9]. Survivors are also at an elevated risk for comorbid conditions, such as cardiovascular disease, osteoporosis, and musculoskeletal impairments, further contributing to the long-term health burden and increased healthcare utilization [10,11]. These cumulative effects highlight the importance of supportive strategies that promote physical and emotional well-being during the survivorship phase.

In addition to their physical and emotional effects, cancer and its treatment impose a significant economic burden. The overall national cost of cancer care in 2015 was estimated at $183 billion and is projected to increase by 34% to $246 billion by 2030, based solely on population growth [12]. These rising costs are compounded by billions of dollars in productivity losses due to disabilities, unemployment, and reduced household productivity [13,14]. Consequently, there is a growing emphasis on modifiable lifestyle behaviors, such as physical activity (PA), which can aid in both cancer prevention and survivorship [15,16,17,18]. This paradigm shift from disease-centered treatment to holistic wellness-oriented care highlights a critical opportunity for oncology care providers (OCPs) and researchers to promote and support healthy lifestyle behaviors among cancer survivors [19,20,21,22].

The American Cancer Society and the American College of Sports Medicine recommend that cancer survivors engage in 150–300 min of moderate-intensity or 75–150 min of vigorous-intensity PA per week, along with muscle-strengthening activities at least twice weekly, tailored to individual needs and health status [23,24]. Extensive evidence demonstrates that PA reduces treatment-related side effects, improves fatigue, mental health, and quality of life, enhances physical function, and reduces recurrence and mortality risk [7,23,25,26]. However, most cancer survivors do not achieve the recommended PA levels, with estimates indicating that fewer than half achieve the recommended PA targets [27,28]. This persistent gap suggests the need to identify determinants of PA engagement to inform scalable and context-sensitive interventions.

Prior research has identified several barriers related to PA among cancer survivors, including treatment-related symptoms, limited guidance from healthcare providers, lack of tailored resources, and challenges with motivation and confidence [22,29,30,31]. However, much of the existing evidence comes from survivors participating in structured lifestyle or exercise interventions, often within academic or clinical settings [32,33], where participants may already be motivated to engage in PA. Consequently, the findings may not fully reflect the experiences of the broader survivor population, particularly those navigating PA outside of structured programs or formal clinical environments. In addition, survivors’ knowledge of PA guidelines and the broader individual, social, and environmental contexts shaping PA engagement have received limited attention, suggesting a limited understanding of how survivors navigate PA in their daily lives [31]. Importantly, several prior studies have not been grounded in a guiding theoretical framework, limiting understanding of how psychosocial, behavioral, and environmental factors interact to influence PA behavior across the survivorship continuum [17,34,35].

These gaps may be particularly salient in predominantly rural states such as Nebraska, where 88 of 93 counties are classified as rural or frontier (<7 person/square mile), and 37% of residents live in these areas [36]. State-level data further suggest the need for intervention, as only 35% of adults meet PA recommendations, while 20% report no leisure-time PA [37]. In geographically dispersed settings, determinants of PA engagement may operate differently due to infrastructure, healthcare access patterns, and community resource distribution [38]. Exploring PA influences within this context allows for a more nuanced understanding of how environmental and behavioral factors intersect in rural cancer survivorship.

To address these gaps, the present study recruited cancer survivors from community-based settings rather than structured exercise or clinical intervention programs and incorporated the Theoretical Domains Framework (TDF), a comprehensive framework synthesizing constructs from 33 behavior change theories [39,40], to examine multilevel determinants of PA among cancer survivors. Using a qualitative approach, this study explored the perceived barriers, facilitators, and recommendations for PA engagement among cancer survivors through the lens of the TDF, thereby providing a theory-informed, contextually grounded understanding of the behavioral and environmental influences that shape PA participation in cancer survivorship.

## 2. Methods

### 2.1. Study Design

This study utilized a phenomenological qualitative design to obtain in-depth insights from cancer survivors regarding their perceptions of the barriers and facilitators of engaging in PA and their recommendations. Phenomenology emphasizes the exploration of lived experiences, and the meanings individuals ascribe to those experiences [41]. Anchored in a social constructivist paradigm, this study sought to understand how participants interpreted their experiences within broader sociocultural and historical contexts [42]. An iterative, interpretive approach was used to capture the participants’ perspectives and develop a comprehensive understanding of their lived experiences [43]. Ethical approval was obtained from the Institutional Review Board of the University of Nebraska Medical Center (IRB# 0468-23-EX).

### 2.2. Sampling and Recruitment

Participants were recruited using purposive and snowball sampling methods to capture diverse perspectives. The eligibility criteria were as follows: (i) age ≥ 19 years, (ii) self-identification as a cancer survivor, (iii) ability to speak and understand English, and (iv) residence in Nebraska. A cancer survivor was defined as an individual with a cancer diagnosis and alive at the time of the study [44]. Participants were excluded if they (i) did not have a cancer diagnosis, (ii) were not residents of Nebraska, or (iii) had cognitive impairment or severe psychiatric conditions that could affect their ability to provide informed consent or meaningfully participate. Efforts were made to ensure diversity in terms of cancer type, age, sex, and race to capture a wide range of opinions, reflecting the experiences of a larger survival community rather than narrowing our insights into a specific sub-group. The planned sample size for the study was 18 cancer survivors. Previous research has shown that 8 to 16 in-depth interviews provide 80–90% data saturation [45,46]. And prior research suggests that conducting 16 interviews will result in 90% data saturation when using thematic analysis [45]. Therefore, after 16 interviews, no additional thematic categories were identified, indicating redundancy and thematic completeness. Saturation was confirmed by the principal investigator (GK) through iterative coding and review of analytic memos, with peer debriefing input from the second coder (PC). Two additional eligible participants who had already expressed interest were interviewed to enhance confirmability; these interviews did not yield new themes. The inclusion of these participants ensured that all interested and eligible voices were represented, and confirmability and depth were added to the thematic analysis.

Recruitment was conducted by sharing a digital flyer with clinical and community partners, including oncology clinics, hospitals, support groups, and survivorship programs, as well as through social media, e-mail distribution, and participant referrals. The flyer provided a brief overview of the study, eligibility criteria, contact information, and a QR code linking to an online screening survey administered through Qualtrics (Qualtrics, Provo, UT, USA). Individuals who completed the screening survey and met the eligibility criteria were contacted by the principal investigator (GK) through their preferred method of communication (e.g., text, email, or phone call) to schedule an interview. Semi-structured interviews were conducted via Zoom 5.10.3 (Zoom Video Communications, Inc., San Jose, CA, USA) at a date and time that was convenient for each participant. A total of 18 cancer survivors were interviewed between August 2023 and January 2024.

### 2.3. Data Collection

The interviews were conducted by the principal investigator (GK), who has approximately nine years of experience conducting qualitative interviews through graduate-level training and prior research [22,47], and received additional mentorship from a faculty advisor (DS), who had over 15 years of qualitative research experience. To evaluate the clarity and relevance of the interview guide and estimate the interview duration, a cognitive interview technique (“think-aloud”) was utilized [48]. Two pilot interviews were conducted prior to data collection, after which minor revisions were made to improve question clarity, for example, explicitly defining the term physical activity (to avoid confusion with the term exercise) and removing redundant items.

Prior to each interview, a consent form outlining the study’s purpose and procedures was emailed to the participants, and verbal consent was obtained at the start of the interview. To minimize interviewer bias and social desirability effects, the interviewer maintained reflexive awareness of potential assumptions related to his background in public health and interest in physical activity. Open-ended, neutrally worded questions were used, and non-directive probes (e.g., “Can you tell me more about that?”) were employed to avoid leading participants. The interviewer avoided affirming or evaluating responses and documented reflexive notes after each interview to monitor potential researcher influence [49,50]. Participants’ sociodemographic and cancer history questions included age, gender, educational level, cancer type, and cancer treatment. A detailed list of questions is provided in a Supplementary File (see Supplementary S1). The interviews ranged from 20 to 60 min (mean = 40 min) in length. Participants received a $50 prepaid Visa gift card as compensation, supported by the University of Nebraska Medical Center College of Public Health Sparks Student Research Award.

### 2.4. Semi-Structured Interview

The interview guide was developed using Theoretical Domains Framework (TDF) version 1 and reviewed by a senior author (DS). TDF v1 synthesizes constructs from 33 behavior-change theories (128 constructs) into the following 12 domains: knowledge; skills; social/professional role and identity; beliefs about capabilities; beliefs about consequences; motivation and goals; memory/attention and decision processes; environmental context and resources; social influences; emotion; behavioral regulation; and nature of the behavior. Each question mapped to at least one TDF v1 domain (see Supplementary File-Supplementary S2) to ensure comprehensive coverage of hypothesized determinants of PA behavior among survivors Although later refinements expanded the TDF to 14 domains, including “optimism” and “reinforcement,” this study explicitly applied the 12-domain v1 because it aligned more closely with the study’s research questions and focus on identifying cognitive, emotional, social, and environmental determinants of physical activity among cancer survivors. The TDF has been validated for behavioral and implementation research and is widely used in qualitative analyses [39].

### 2.5. Data Analysis

The interviews were audio-recorded, professionally transcribed verbatim, and de-identified prior to analysis. The transcripts were uploaded into MAXQDA 2024 (VERBI Software, Berlin, Germany) for data management and analysis. Data were analyzed using a deductive-inductive thematic analysis approach. Although the TDF informed the development of the interview guide (deductive), coding and theme development were conducted inductively, allowing themes to emerge directly from participants’ narratives rather than being constrained by predefined theoretical categories.

This study employed thematic analysis while drawing on principles of framework analysis, following the five iterative steps outlined by Gale and colleagues [51]: (1) familiarization with the data, (2) identifying an initial coding framework, (3) indexing transcripts systematically, (4) charting data into thematic matrices, and (5) mapping and interpreting patterns across participants. The principal investigator (GK) reads each transcript multiple times to gain immersion and identify significant statements that captured key meanings. These statements were grouped and compared across transcripts to identify recurring patterns and develop preliminary categories for analysis.

To ensure study rigor, credibility, and trustworthiness, we incorporated peer debriefing and triangulation throughout the analytic process [52]. A preliminary codebook was initially informed by broad topic areas from the semi-structured interview guide; however, during coding, new codes were added inductively based on participant responses, allowing the codebook to be refined. Two researchers (GK and PC) independently coded five randomly selected transcripts (~25% of the dataset) and compared the coding decisions to assess consistency. Discrepancies were discussed until a consensus was reached, after which the principal investigator (GK) continued coding the remaining transcripts.

Throughout the coding process, analytic memos and an audit trail were maintained to support transparency and reflexivity [53]. After coding was completed, themes and subthemes were identified, refined, and organized to represent coherent patterns in the participants’ experiences. Direct quotations were selected to illustrate the thematic interpretation [54]. Themes were then mapped back to the relevant domains of the TDF during the interpretive phase, providing a theoretical context while preserving the inductively generated nature of the findings. This study followed the Standards for Reporting Qualitative Research (SRQR) framework [55].

## 3. Results

### 3.1. Sample Characteristics

Eighteen cancer survivors participated in the semi-structured interviews (Table 1). More than two-thirds of the participants were female and married, and half were non-Hispanic whites. The mean age for cancer diagnosis was 60.2 years (SD ± 11.4). The cancer diagnoses included a variety of types, with breast cancer being the most common (44.4%). Cancer stages ranged from I to IV, with half being stage III, and treatments included surgery, adjuvant and neoadjuvant chemotherapy.

### 3.2. Qualitative Findings

Guided by the 12-domain TDF v1 [39], thematic analysis revealed three overarching themes: barriers, facilitators, and recommendations, which encompassed multiple behavioral determinants of PA among cancer survivors. These themes reflected a dynamic interaction among individual, social, clinical and environmental influences and aligned with all twelve TDF domains (Table 2).

### 3.3. Theme 1: PA Barriers Faced by Cancer Survivors

Qualitative analysis identified multiple barriers that limited cancer survivors’ ability to engage in regular PA. These barriers clustered across four interrelated levels: individual, social, clinical and environmental barriers, each encompassing specific challenges faced by cancer survivors.

#### 3.3.1. Individual-Level PA Barriers

Cancer survivors described individual-level barriers including low motivation, limited awareness of PA guidelines, competing demands, and treatment-related physical limitations. Collectively, these barriers can make it daunting for survivors to initiate or maintain regular PA.

Lack of Motivation and self-efficacy as barriers to PA: Cancer survivors described low motivation as a key barrier to PA, often linked to treatment-related fatigue and emotional burden (n = 14). One shared, *“Most of the time, I get lazy … I am not motivated” [P01]*, while another acknowledged, *“We have a treadmill … I just need to use them” [P11]*. Others recognized internal resistance, stating, *“What prevents me sometimes is myself-just not wanting to do it” [P03].*

Limited awareness of PA guidelines: Cancer survivors (n = 16) were unfamiliar with formal PA recommendations for cancer survivors, often responding, *“No, I don’t know” [P02].* Some survivors described relying on self-directed approaches to wellness rather than evidence-based guidance. As one survivor noted, *“No, I have my own recommendations I follow. So, I don’t know” [P10].*

Competing demands and limited time as barriers to PA: Time emerged as another barrier to engaging in PA (n = 15), with some survivors describing the challenge of balancing professional, familial, and personal responsibilities. One participant noted, *“Sometimes I have to stay late for meetings… Then I can’t do the walking that I intend to do. Scheduling can be a hindrance” [P05].* Another shared, *“It’s just time because I work full time. I’m a single parent, and I have a part-time job every other weekend” [P14]*.

Physical limitations from treatment side effects and comorbidities: Cancer survivors described treatment side effects and comorbid conditions that restricted their ability to engage in PA (n = 16). One survivor shared, *“Chemotherapy was really hard for me … There were days I did not get out of bed” [P01].* Another participant noted reduce activity due to persistent pain and fatigue, *“I do less activity now due to fatigue and muscle aches and pains” [P09].* Neuropathy and surgical limitations further restricted PA, with one participant noting, *“The neuropathy in my feet has made me fear falling during physical activity” [P16]*, and another stating, *“I had one of the lobes of my lung removed … That limits me” [P12].*

#### 3.3.2. Social-Level PA Barriers

Social barriers highlight the importance of a supportive network in facilitating PA among cancer survivors. The lack of social support from family, friends can significantly diminish their motivation and opportunities for engaging in PA.

Limited social in PA engagement: Cancer survivors described a lack of encouragement and companionship from family and friends as a barrier to maintaining PA (n = 13). One survivor shared, *“I really don’t have anybody … I always say I’m alone, but I’m not lonely” [P13],* while another noted, *“I don’t have anyone that says, ‘Come on, let’s go for a walk or a jog’” [P06]*. Relocation and disrupted social networks further limited support, with one participant explaining, *“I don’t have many friends in Omaha yet … I don’t know anybody because of cancer treatment” [P09].*

#### 3.3.3. Clinical-Level PA Barriers

Cancer survivors described structural and communication-related gaps within oncology care that limited their engagement in physical activity. These barriers reflected missed opportunities for tailored counseling and integration of PA guidance into survivorship care. Cancer survivors reported receiving minimal or nonspecific exercise guidance following treatment (n = 16). One participant stated, *“Not for my cancer” [P02],* and another shared, *“Not directly from my healthcare providers-they kind of mentioned it would be a good idea, but there was no specific guidance” [P15].* Limited clinic time further restricted PA counseling, with one participant noting, *“I saw an oncologist… but only for five minutes. They’re so busy; they don’t have time to talk to me (about PA)” [P08].* Some expressed frustration with the lack of guidance, stating, *“I think they just want to give you a blanket and tell you to lay down the rest of your life” [P17].* Missed referrals to supportive programs were also evident: *“She never said anything about the YMCA Livestrong program or the American Cancer Society” [P11].*

#### 3.3.4. Environmental-Level PA Barriers

Environmental barriers present additional obstacles to PA among cancer survivors. Accessibility issues, such as the limited availability of parks, trails, and safe sidewalks and concerns about safety, finances, and transportation, can restrict their ability to engage in outdoor activities.

Limited accessibility as barriers to PA resources: Cancer survivors frequently cited a range of environmental and resource-related limitations that hindered their ability to engage in regular PA (n = 14). A lack of safe, pedestrian-friendly infrastructure was a common concern with one participant noting, *“There are no sidewalks where I live … But that will never happen there.”* Others emphasized how distance and traffic limited their access to fitness facilities difficult, as one participant noted, *“I wish the YMCA was closer. I don’t like to drive in Omaha traffic” [P04].* Financial barriers added to the challenge *“I got a scholarship for a year because I was in LIVESTRONG, but $70 a month was a bit much after paying all these medical bills” [P12].* Additionally, the discontinuation of suitable exercise classes frustrated some survivors who made efforts to integrate PA into their schedules, *“On the day that I made time to do the total body conditioning … they don’t offer that class anymore” [P10].* Safety concerns, particularly for women, further restricted outdoor activity options: *“There’s a park not too far from our house… I don’t know if I feel safe to go there,”* and *“I wouldn’t walk far at night; in the winter it gets dark sooner. I’m more careful then, especially as a woman” [P14].*

Weather and seasonal conditions as barriers to PA: Weather emerged as a prominent environmental barrier that limited cancer survivors’ ability to maintain consistent regular PA throughout the year (n = 13). Harsh winters characterized by ice, snow, and cold temperatures often discourage outdoor activities. One participant noted, *“Winter, you know—ice, snow, those kinds of things … that would prevent somebody from enjoying a few hours on a bicycle outside” [P01].* Others described avoiding activity during extreme cold: *“If it’s just been too cold, I don’t want to walk around when the air hurts my face” [P16].* Conversely, high temperatures also posed significant challenges, especially for those managing treatment-related heat sensitivity or medication restrictions: *“The weather-it was so hot, hot flashes. I can’t walk in the heat,”* and *“I don’t do too well in heat … I’m not supposed to be out in the sun too long” [P05].* Many participants summarized the struggle as a year-round challenge, with one stating, *“Welcome to Nebraska! We’re very limited … more active in spring and summer, less in fall and winter. The weather has a lot to do with it” [P18].*

### 3.4. Theme 2: Facilitators of PA

While numerous barriers hindered consistent PA among cancer survivors, several facilitators emerged that enabled engagement and sustained participation. These facilitators spanned individual, social, and environmental levels.

#### 3.4.1. Individual-Level Factors

Adequate knowledge-related PA: Cancer survivors conceptualized PA in diverse and personally meaningful ways (n = 16). For some, it meant *“just staying active over time, as much as possible” [P03],* emphasizing consistency over intensity. Others associated it with specific recreational or outdoor activities like *“bicycling, walking, hiking, and alpine skiing” [P07],* as well as structured and everyday movement, including *“going to the gym … dancing,* *walking, or even yard work” [P12]*. Several defined PA broadly as avoiding sedentary behavior, noting, *“Not sitting… leaving the house? Running errands?” [P01].* As one participant summarized, *“It’s not about sweating in the gym-it’s about walking around the park, just getting my heart rate up a little bit” [P05].*

Motivation for PA: Cancer survivors described strong intrinsic motivation driven by emotional and physical rewards (n = 9). Many emphasized the positive effects of PA, noting, *“It makes me feel really good … everything is just better” [P06]*. Commitment to routine also supported engagement, with one participant stating, *“At least 30 min … I try not to skip Saturday and Sunday” [P10].* Others described determination despite barriers: *“I make time no matter what” [P01].* For some, surviving cancer itself strengthened motivation: *“Being alive has made me want to step up” [P07].*

Goals for PA: Participants described diverse goals reflecting recovery stage and personal priorities(n = 7). Some focused on small daily achievements such as *“drink water, get out of bed, go downstairs” [P10]*, while others pursued structured routines including *“walk five miles … and do yoga” [P02]* or quantifiable targets like *“10,000 steps per day” [P08]*. Several emphasized self-accountability, noting, *“I signed a contract with myself” [P18]*, and a desire to regain prior fitness levels.

Perceived Health Benefits of PA: Cancer survivors described PA as essential to their recovery, reporting improved strength, energy, and resilience to treatment effects (n = 7). One participant shared, *“It makes you healthier to fight cancer” [P04],* while another emphasized, *“Anytime your physique is optimal … your blood is flowing, your heart is pumping…you’re a stronger person” [P07].*

Survivors emphasized the mental health benefits of regular PA, describing improved mood and emotional well-being. As one participant noted, *“It’s also helped my mental health” [P06].* Some attributed these effects to increased positivity and endorphin release, with one explaining, *“If you’re happy, you release endorphins … I think they have healing power” [P03].*

Beyond immediate benefits, survivors linked PA to symptom management and long-term health outcomes. One explained, *“Having cancer in the lungs, I feel that keeping my lungs moving will help me in the long run” [P12].* while others believed regular PA contributed to reduced recurrence risk and extended survival, with one participant noting, *“It helps with health outcomes, reducing recurrence, and living longer” [P08].*

#### 3.4.2. Impact of Social Support on PA

Social support emerged as a significant facilitator of physical activity among cancer survivors, influencing motivation, consistency, and overall well-being through various relationships and channels

Family members: Emerged as a key facilitator of PA among cancer survivors, providing encouragement, companionship, and practical assistance (n = 11). Many survivors described family involvement as both inspirational and participatory. One shared, *“I ride a bicycle with my uncle once a week … my 80-year-old uncle and I ride 20 miles in a week” [P05],* reflecting both support and shared commitment. While another drew motivation from daily gestures, one said, *“My wife supports me and participates. When I leave in the morning, she always tells me she’s proud of me for getting up and going” [P10].*

Partners who were physically active often served as role models, encouraging shared pursuits such as walking or hiking, and some benefited from expert advice within the family. One participant said, *“My sister is a professional bodybuilder, and her husband, a personal trainer, always says I need to do weights every day” [P01].* Others mentioned tangible support, such as helping purchase exercise equipment, indicated the practical ways family fosters PA, *“My husband’s the one who said we needed to buy that exercise bike” [P18].*

Friends and co-workers: Valued as vital sources of companionship, motivation, and accountability for engaging in PA, alongside family support (n = 7). Social interactions often transformed PA into an enjoyable and therapeutic experience as one survivor shared, *“A neighbor actually invited me to go for walks. We started slowly, and not only were we walking, we were talking, so it was almost like our physical and mental therapy” [P06].* Another emphasized the joy of shared activity, *“When I was walking with friends, it was very enjoyable because I really liked her company… we both felt great and looked forward to the next day” [P09].*

Further, coworkers were also mentioned as motivators: *“I have a really good friend here at work. He’s starting to exercise as well. And we just have the camaraderie back and forth to motivate each other” [P12].* Participants in group activities and yoga communities also provided ongoing encouragement and accountability, *“There’s a group of women, and if you miss too many weeks, they ask, ‘Where were you?” [P15]* and *“I have a great yoga Sangha community that sent me videos when I was in treatment” [P13].*

#### 3.4.3. Clinical-Level PA Facilitator

Cancer survivors identified oncology care providers as important facilitators of PA when clear and personalized guidance was provided, highlighting the influence of clinical endorsement on survivors’ confidence and engagement in PA. Survivors described benefiting from recommendations and encouragement tailored to their unique condition and capabilities (n = 4). One participant shared, *“The oncologist said walking would be my best physical activity” [P02]*, highlighting the practical guidance received. Another noted, *“She (oncologist) was encouraging water aerobics for my right arm and just like being gentle. She really encouraged the gentle water aerobics yoga” [P11].*

#### 3.4.4. Environmental Factors

Environmental factors played an important role in shaping cancer survivors’ ability to engage in physical activity, with weather conditions and the availability of community resources emerging as key facilitators of participation.

Weather: Many (n = 13) described how favorable weather encourages participation in activities such as walking in local parks, with one survivor noting, *“My husband and I go to the park that’s real close to our house, and we work the walking trail there in the summertime and when weather is favorable” [P05].* The enjoyment of spending time outdoors was further reflected by another survivor who found both joy and engagement in gardening during nice weather: *“That’s the good thing when it is nice, I like to be outside. I had a garden outside. I mean, it’s nice to go out and, you know, tend to my flowers in the garden” [P10].* Survivors also discussed adapting PA to the seasonal changes and less favorable conditions. When outdoor walking or running was less practical due to cold or snowy weather, participants found alternative ways to stay active such as raking leaves and shoveling snow. As one participant noted, *“But when it snows around here, or when it’s fall time, I’m raking leaves or helping to shovel snow. So, there’s always something that needs to be done” [P18].*

Availability of PA resources in the community: Cancer survivors emphasized the value of accessible community resources in supporting and motivating their participation in PA (n = 7). Programs such as the YMCA’s LIVESTRONG program and Time to Heal were frequently praised for providing structured support tailored to survivors’ needs. One survivor shared, *“Yes, I’ve gone to the YMCA, and they have a Livestrong program. And that was amazing” [P04].* Survivors also appreciated the diversity and affordability of options available within their community, noting, *“The YMCA actually offers very affordable personal training and a ton of different programs” [P16],* emphasizing accessibility and adaptability. In addition to these programs, other resources, including SilverSneakers and local facilities such as the generation center, were also mentioned as sources of motivation and regular encouragement. As one survivor described, *“I joined SilverSneakers, so they send regular emails with how important physical activity is, and they recommend (PA), you know, by age group, male, female, kids, like how much and for how long” [P07].*

### 3.5. Theme 3: Recommendations to Enhance PA Engagement

Cancer survivors provided practical recommendations to enhance PA participation, emphasizing the importance of enjoyable and accessible activities, stronger educational and community support, and more personalized guidance from oncology care providers.

Cancer survivors: Cancer survivors emphasized the importance of simple, accessible, and enjoyable forms of PA, particularly walking, as effective and manageable strategies to maintain health and support recovery (n = 15). One participant shared, *“Walking is one of the best, easiest ways to exercise-you don’t need to join a gym, just walk every day” [P03].* Survivors also mentioned the role of enjoyment and social motivation in sustaining engagement, with advice such as *“Keep moving, get in a group that motivates you, and have fun-the biggest part is to have fun doing it” [P06].* In addition to these motivational factors, survivors expressed a strong need for expanded educational and community resources to support their ongoing PA effort, asking for *“more pamphlets, more information, more support groups” [P14].* Structured and community-based programs like the YMCA LIVESTRONG initiative were highly valued for their tailored approach and affordability: *“I’d really recommend the YMCA LIVESTRONG program-it’s awesome that they offer that for free” [P11].*

Oncology care providers: Cancer survivors (n = 16) expressed the desire for clear, practical advice tailored to their PA and unique health needs: *“I think you’re more likely to get guidelines from your oncologist-to say, don’t do this activity, but it’s okay to do that one” [P02].* The value of group-based recommendations was highlighted as a means to boost motivation and provide social support, as one noted, *“I wish they would tell people to join a group setting where they can have friends to keep them motivated” [P13].* Several participants highlighted missed opportunities for referral to existing programs, such as the YMCA’s LIVESTRONG initiative: *“They never even mentioned the YMCA LIVESTRONG program… every oncology doctor should mention that to every patient” [P11].* Survivors further suggested the importance of initiating conversations about PA should begin early in treatment: *“Maybe if it was brought up sooner while I was going through chemo, that would help more” [P01].*

## 4. Discussion

To our knowledge, this is among the first qualitative studies conducted in the U.S. Midwest to apply the TDF in exploring cancer survivors’ perspectives on barriers, facilitators, and recommendations for PA. This study provides valuable insights into the multilevel factors such as individual, social, clinical and environmental that shape PA behaviors among survivors, within the predominantly rural Midwestern context of Nebraska, where geographic dispersion and healthcare centralization may influence access to supportive resources. The findings indicate the complex and interdependent nature of these influences, highlighting how personal motivation, social support, and environmental accessibility collectively determine survivors’ ability to engage in and sustain PA participation.

At the individual level, participants identified major barriers, including a lack of motivation, limited awareness of PA guidelines, time constraints, and physical limitations associated with treatment side effects and comorbidities. These findings are consistent with prior research demonstrating that cancer survivors face distinctive challenges in maintaining physical activity, despite its known benefits for recovery and long-term survivorship [27,56]. The pervasive lack of motivation aligns with previous work [31,57,58] and appears to be driven largely by fatigue and emotional exhaustion resulting from cancer treatment. Evidence suggests that interventions incorporating motivational interviewing and goal setting can effectively enhance engagement and activity levels [59,60]. Moreover, digital health technologies such as mobile apps and wearable devices could offer continuous feedback and individualized support, potentially mitigating motivational barriers [61,62], particularly in geographically dispersed settings such as Nebraska, where access to structured, in-person programs may be constrained by distance and transportation demands.

Our study showed a notable lack of knowledge about PA guidelines for cancer survivors, consistent with the findings of previous studies [56,63,64], which highlighted a general lack of awareness and comprehension of these guidelines. Targeted educational initiatives that provide clear, accessible, and customized PA information are crucial to bridging this gap [24,65]. These programs must be incorporated into survivorship care plans and endorsed by cancer care professionals, who have a vital role in spreading this knowledge [22]. Moreover, developing patient-centric materials like easy-to-navigate websites or pamphlets with precise PA guidelines for cancer survivors could help reduce information-related barriers [24,66], particularly in Nebraska, where oncology services are often centralized in urban areas, potentially limiting follow-up lifestyle counseling for survivors residing in rural counties.

Cancer survivors face difficulty finding time for PA due to work and family responsibilities, consistent with other research studies [27,56,67,68]. To overcome this obstacle, it is necessary to implement adaptable and customized strategies that align with the survivors’ current schedules and obligations [69]. Implementing strategies like time management workshops, adaptable exercise regimens, and encouraging brief periods of PA during the day can provide practical solutions. Employer-backed programs offering flexible work hours or wellness efforts could help reduce time-related obstacles for employed survivors [70,71], particularly in rural regions where travel time for employment or healthcare may further compress available time for structured PA.

Cancer survivors sometimes face significant challenges to engage in PA due to physical restrictions such as pain, exhaustion, and treatment of side effects. The results align with recent studies [17,27,31,56], which emphasized the influence of physical side effects on activity levels. Rehabilitation programs that include physical therapy, pain management, and adaptive PA interventions are crucial for overcoming these obstacles [72,73]. It is essential to create fitness programs that cater to the unique physical restrictions of cancer survivors, including low-impact workouts and personalized strength training [17,24,32,72,73,74], especially in rural settings where access to specialized cancer rehabilitation services may be limited.

At the social level, the study underscores the vital role of family, friends, and healthcare providers in promoting PA. Many participants described limited encouragement from family or friends, often leading to feelings of isolation. This aligns with existing research, emphasizing that the quality of social interactions is critical for sustaining engagement in PA [75,76]. Similarly, inadequate support from oncology care providers mirrors earlier findings [22,64,77], underscoring the need for consistent PA counseling within oncology and survivorship care. Participants’ perception that the transition to survivorship lacked sufficient guidance highlights the importance of integrating structured discussions on PA early in the care continuum, particularly in Nebraska, where centralized oncology services may reduce opportunities for repeated lifestyle counseling among survivors residing in rural and frontier counties.

Barriers related to the environment include difficulties in accessing outdoor activities, financial limitations, and weather-related hindrances. Access to safe and convenient places for PA, such as parks, trails, and fitness centers, is a significant obstacle. As criticized by survivors, the absence of pedestrian-friendly infrastructure highlights a larger problem in urban planning and community design that does not promote active lives [78]. Distance and transportation issues may hinder access to fitness centers, which can discourage consistent PA [35,56,67]. Financial difficulties worsen this issue, as the expense of a gym membership becomes a considerable burden, particularly when combined with increasing medical expenses [27,79]. In Nebraska, where nearly half of the counties are classified as rural and many as frontier, limited pedestrian infrastructure and long travel distances may further constrain consistent PA engagement.

Safety concerns, particularly in public spaces or at night, significantly affect cancer survivors’ willingness to participate in outdoor physical activities [27,78,80]. These challenges are particularly noticeable for women and underscore the importance of having safe, well-lit, and easily accessible areas for PA [81]. Implementing community watch programs and enhancing lighting in public parks and trails can improve safety perceptions. Organizing group walks or exercise classes in public spaces might improve feelings of safety and community, as suggested by previous studies [75,78,81].

Harsh temperatures and severe weather conditions pose significant challenges to outdoor PA. These challenges are particularly evident in regions with varying temperatures, where seasonal changes can significantly affect the feasibility of outdoor exercise [56,78]. In states such as Nebraska, characterized by marked seasonal variation and severe winter conditions, weather-related barriers may substantially disrupt consistent outdoor PA. Supporting and broadening indoor PA choices, such as mall walking programs or indoor swimming, can provide alternatives during extreme weather conditions [82]. Developing exercise routines adapted to various weather conditions, including online or home-based options, can support the continuity of PA throughout the year [83,84].

### Strengths and Limitations

One strength of this study is the systematic and theory-informed approach used in data collection and analysis to enhance the rigor and trustworthiness of the analysis: checking transcripts against audio recordings and field notes taken, and triangulation among coders by consensus to ensure rigor. Purposeful sampling was used to select a wide range of survivors with different experiences. We chose one-on-one interviews to focus on each participant’s ideas and experiences during the interview. This strategy was adopted to ensure that individuals’ distinctive viewpoints were not impacted by the dynamics of group debates or worries about their ideas being regarded as ‘incorrect’ by others [85].

Despite its strengths, this study had several limitations that should be considered when interpreting the findings. First, the sample included only cancer survivors from one Midwestern state. Therefore, the findings may not be transferable to cancer survivors in different regions of the United States and other countries, as PA behavior may vary. Additional research is needed to determine whether these findings can be replicated elsewhere. Although the sample included individuals from multiple racial and ethnic backgrounds, the study was not designed to examine differences in barriers across specific racial/ethnic groups. Similarly, while participants represented a range of cancer types and stages, comparisons by diagnosis, treatment intensity, or disease severity were beyond the scope of this analysis. Recruitment through digital flyers and social media may have attracted survivors who were more health-aware, motivated to discuss physical activity, or comfortable with digital technologies, potentially underrepresenting those who are less active, less technologically literate, or less connected to survivorship resources. Social desirability bias should be acknowledged, as cancer survivors may have responded to appease the researchers. However, steps were taken to reduce biases by establishing rapport before the interviews, asking probing questions, and noting whether participants gave potentially socially desirable responses.

## 5. Conclusions

This study advances understanding of the multilevel determinants of PA among cancer survivors, demonstrating that engagement is shaped by interconnected influences across individual, social, clinical and environmental levels. At the individual level, survivors described treatment-related symptoms, fatigue, limited motivation, time constraints, and gaps in awareness of PA guidelines as key challenges. At the clinical level, insufficient counseling and encouragement from oncology care providers influenced participation, while at the environmental level, weather conditions and limited access to safe and affordable resources further constrained engagement. By applying the TDF, this study provides a structured, theory-informed perspective on how knowledge, beliefs, social support, clinical practices, and contextual factors interact to shape PA behavior during survivorship. These findings highlight the importance of integrating tailored PA education, proactive and individualized exercise counseling, and accessible community- or home-based programs into survivorship care models. Theory-driven, patient-centered strategies are essential to promote sustainable PA engagement and improve long-term survivorship outcomes.

## Figures and Tables

**Table 1:** Descriptive characteristics of the cancer survivors in the study (n = 18).

Characteristics	N (%)
Age (years), mean ± SD (range)	60.2 ± 11.4 (41–82)
Years since diagnosis, mean ± SD (range)	8.6 ± 10.8 (1–42)
Gender	
Male	4 (22.2)
Female	14 (77.8)
Race/Ethnicity	
Non-Hispanic White	9 (50.0)
Non-Hispanic Black	4 (22.2)
Hispanic	3 (16.7)
Asian	2 (11.1)
Marital Status	
Married/living with partner	14 (77.8)
Divorced or single	3 (16.7)
Widow/widower	1 (5.6)
Education Level	
Vocational school/Some college	7 (38.9)
Associate	3 (16.7)
Bachelor	2 (11.1)
Masters	1 (5.6)
Professional	5 (27.8)
Employment Status	
Employed full-time	6 (33.3)
Employed part-time	2 (11.1)
Retired	8 (44.4)
Medical Leave	1 (5.6)
Homemaker	1 (5.6)
Annual Income ($)	
<25,000	2 (11.1)
25,000–50,000	2 (11.1)
50,000–75,000	5 (27.8)
≥75,000	9 (50.0)
Insurance Type	
Medicare/Supplement	7 (38.9)
Private	11 (61.1)
Smoking Status	
Current	1 (5.6)
Former	5 (27.8)
Never	12 (66.7)
Alcohol consumption	
Current/Occasional	12 (66.6)
Former	3 (16.7)
Never	3 (16.7)
Co-morbidities	
Yes	10 (55.5)
No	8 (44.5)
Cancer Diagnosis	
Breast	8 (44.4)
Lung	1 (5.5)
Colorectal	2 (11.1)
Prostate	2 (11.1)
Hodgkin Lymphoma (Hodgkin disease)	1 (5.5)
Bone	1 (5.5)
Kidney	1 (5.5)
Vulvar	1 (5.5)
Peritoneal Papillary serous	1 (5.5)
Cancer Stage	
I	1 (5.5)
II	5 (27.8)
III	9 (50.0)
IV	3 (16.7)
Cancer Treatment	
Adjuvant chemotherapy	7 (38.8)
Neoadjuvant chemotherapy	6 (33.3)
Others *	5 (27.9)

* Others include (one each of Chemotherapy and Radiation, Hormone, Lumpectomy, Nephrectomy, Intravesical Therapy, Chemotherapy, and Stem cell transplant).

**Table 2:** Themes and subthemes related to physical activity among cancer survivors, mapped to the 12 domains of the Theoretical Domains Framework (TDF v1).

Themes	Level	Subthemes	Aligned TDF v1 Domains
Barriers	Individual Factors	Lack of motivation and self-efficacy, limited awareness of PA guideline, time constraints, and physical constraints due to treatment effects and comorbidities.	Knowledge; Motivation & Goals; Beliefs about Capabilities; Emotion; Memory, Attention & Decision Processes; Nature of Behavior
	Social Factors	Limited support from family, and friends.	Social Influences; Emotion
	Clinical Factors	Limited PA guidance from oncology care providers	Social/Professional Role & Identity
	Environmental Factors	Limited resource accessibility and unfavorable weather.	Environmental Context & Resources
Facilitators	Individual Factors	PA knowledge, PA motivation, PA Goals and health benefits.	Knowledge; Motivation & Goals; Beliefs about Consequences; Behavioral Regulation; Nature of Behavior
	Social Factors	Support from family, and friends.	Social Influences; Social/Professional Role & Identity; Emotion
	Clinical Factors	Personalized PA recommendations	Social/Professional Role & Identity; Skills; Behavioral Regulation
	Environmental Factors	Weather and PA resources availability.	Environmental Context & Resources
Recommendations	Cancer Survivors	Desire for more PA education and individualized programs; preference for enjoyable, flexible, and goal-oriented PA options	Knowledge; Skills; Motivation & Goals; Behavioral Regulation
	Oncology Care Providers	Suggested improved PA counseling, resource sharing, and integration of exercise referrals into survivorship care plans	Social/Professional Role & Identity; Environmental Context & Resources; Skills

Note: PA = physical activity; TDF = Theoretical Domains Framework (v1).

## Data Availability

The de-identified qualitative data supporting the findings of this study are available from the corresponding author, Gaurav Kumar (gaurav-kumar-3@ou.edu), upon reasonable request and are subject to Institutional Review Board restrictions on confidentiality.

## Supplementary Materials

The following supporting information can be downloaded at: https://www.mdpi.com/article/10.3390/cancers18050817/s1. Supplementary S1: Socio-demographic questionnaire for Cancer survivors; Supplementary S2: Semi-structured questions based on the Theoretical Domains Framework (TDF) for the Cancer Survivors.
