# Supplementary material for: Perceived Barriers and Facilitators to Physical Activity Engagement Among Cancer Survivors: A Qualitative Study
Source: Cancers (Basel). 2026 Mar 3;18(5):817. doi: 10.3390/cancers18050817 (PMC12984601; doi:10.3390/cancers18050817)
Supplement: Supplementary file 1 [file cancers-18-00817-s001.zip › cancers-4114184-supplementary.pdf]

Supplementary S1: Socio-Demographic Questionnaire for Cancer Survivors

1. **Age (Years)**

    - ☐ 18-39
    - ☐ 40-49
    - ☐ 50-59
    - ☐ 60-69
    - ☐ 70-79
    - ☐ 80+

2. **Gender**

    - ☐ Male
    - ☐ Female

3. **Race/Ethnicity**

    - ☐ Non-Hispanic White
    - ☐ Non-Hispanic Black
    - ☐ Hispanic
    - ☐ Others (Specify) _____________

4. **Marital status**

    - ☐ Single
    - ☐ Married/Living with a partner

☐ Divorced/Separated

☐ Widowed

5. **Employment status**

☐ Employed full-time

☐ Employed part-time

☐ Unemployed

☐ Retired

☐ Homemaker/Medical leave

6. **Educational level**

☐ High School Diploma or below

☐ Vocational/Trade School

☐ Some college

☐ Associate degree

☐ Bachelor’s degree

☐ Graduate School

7. **Household income**

☐ Less than $25,000

☐ $25,000 – 49,999

☐ $50,000 – 74,999

- ☐ $75,000 and more

8. **Zip code of residence** ______________

9. **Health Insurance**

   - ☐ Private
   - ☐ Medicare
   - ☐ Medicaid
   - ☐ No insurance
   - ☐ Others (Specify) ___________

10. **Smoking Status**

    - ☐ Never
    - ☐ Former smoker
    - ☐ Current smoker

11. **Alcohol consumption status**

    - ☐ Never
    - ☐ Former drinker
    - ☐ Current drinker

12. **Co-morbidities**

    - ☐ Yes (Specify)_____________
    - ☐ No

13. **Type of Cancer** (Specify)________

14. **Cancer stage**

- ☐ 0
- ☐ I
- ☐ II
- ☐ III/IV

15. **Cancer treatment**

- ☐ Lumpectomy
- ☐ Lumpectomy with dissection
- ☐ Radiation
- ☐ Chemotherapy
- ☐ Hormone treatment
- ☐ None

16. **Years since diagnosis ________**

Supplementary S2: Semi-structured questions based on The Theoretical Domains Framework (TDF) for the Cancer Survivors

Knowledge and Beliefs:	• What comes to mind when you hear the term "physical activity"?<br>• Do you believe physical activity can help manage or recover from cancer? Why or why not?<br>• Do you know any physical activity recommendations?<br>• What is the most trusted source of PA information? Do you also search for PA information from online platform (google, Facebook group) and how it has changed your PA.
Memory and Emotion:	• Can you explain a time in which you most enjoyed being physically active?<br>• How often do you do that type of activity currently?<br>• If not, what keeps you from doing that specific activity today?
Nature of Behavior:	• Has your physical activity level changed since your cancer diagnosis? If yes, how has it changed?<br>• How many minutes of physical activity would you get in a week?<br>• How does it differ between weekdays and weekends, time of day?
Motivation & Goals:	• Do you currently have any physical activity goals for yourself? Please explain.<br>• Are there any factors that motivate or prevent you from achieving your goal?
Skills:	• Are there any specific skills or techniques that you would like to learn or improve to enhance your physical activity as a cancer survivor?
Environmental Context and Resources:	• What resources or facilities are available to support your physical activity as a cancer survivor?

	• Is anything specific within your community that you use?<br>• Are there any environmental factors that facilitate or hinder your physical activity, such as access to parks, safe walking paths, or weather?
Social/Professional Role & Identity; Social Influence:	• Who in your social circle (family, friends, support groups) encourages or supports your engagement in physical activity as a cancer survivor? How they do it. Can you give me some examples.<br>• Have you received any advice or recommendations from healthcare providers regarding physical activity after cancer?
Behavioral regulation:	• What barriers do you have to be physically active?<br>• What do you think could help you to overcome these barriers?

Additional questions

- What would you recommend to other cancer patients or survivors related to physical activity or oncology care providers.
- In your opinion what would be your preference of PA intervention. Eg: in-person, digital, alone or group based.
